# Molecular Docking and Molecular Dynamics Simulations Discover Curcumin Analogue as a Plausible Dual Inhibitor for SARS-CoV-2

**DOI:** 10.3390/ijms23031771

**Published:** 2022-02-04

**Authors:** Shailima Rampogu, Gihwan Lee, Jun Sung Park, Keun Woo Lee, Myeong Ok Kim

**Affiliations:** 1 Division of Life Sciences, Division of Applied Life Science (BK21 Plus), Research Institute of Natural Science (RINS), Gyeongsang National University (GNU), 501 Jinju-daero, Jinju 52828, Korea; shailima.rampogu@gmail.com (S.R.); pika0131@naver.com (G.L.); 2 Division of Life Science and Applied Life Science (BK21 FOUR), College of Natural Sciences, Gyeongsang National University, Jinju 52828, Korea; jsp@gnu.ac.kr

**Keywords:** natural compound analogues, main protease, SARS-CoV-2, DDX3, dual inhibitor

## Abstract

Recently, the world has been witnessing a global pandemic with no effective therapeutics yet, while cancer continues to be a major disease claiming many lives. The natural compound curcumin is bestowed with multiple medicinal applications in addition to demonstrating antiviral and anticancer activities. In order to elucidate the impact of curcumin on COVID-19 and cancer, the current investigation has adapted several computational techniques to unfold its possible inhibitory activity. Accordingly, curcumin and similar compounds and analogues were retrieved and assessed for their binding affinities at the binding pocket of SARS-CoV-2 main protease and DDX3. The best binding pose was escalated to molecular dynamics simulation (MDS) studies to assess the time dependent stability. Our findings have rendered one compound that has demonstrated good molecular dock score complemented by key residue interactions and have shown stable MDS results inferred by root mean square deviation (RMSD), radius of gyration (Rg), binding mode, hydrogen bond interactions, and interaction energy. Essential dynamics results have shown that the systemadapts minimum energy conformation to attain a stable state. The discovered compound (curA) could act as plausible inhibitor against SARS-CoV-2 and DDX3. Furthermore, curA could serve as a chemical scaffold for designing and developing new compounds.

## 1. Introduction

Herbal medicines are foremost in providing new antiviral sources, and discovering the antiviral mechanisms of these natural products has revealed their interaction point within the life cycle of the virus [1]. Turmeric (Curcuma longa) belongs to the ginger family, and has been used as a food preservative and a natural medicine [2]. The essential elements of turmeric are the curcuminoids that infer a distinct yellow colour [2]. The major constituents of curcuminoids that exists in turmeric are demethoxycurcumin, cyclocurcumin, bisdemethoxycurcumin, and the curcumin comprising of 2–5% of the spice [2]. Curcumin is crucial in inhibiting viral infections, adapting various mechanisms either by impeding the viral replication or by repressing the signalling pathways involved in viral replications [3]. 

Severs acute respiratory syndrome coronavirus-2 (SARS-CoV-2) is responsible for Coronavirus disease 2019 (COVID-19), which has been causing a serious health emergency across the world [4], and was initially noticed in China [5]. CoVs belong to a single-stranded RNA viruses family, capable of infecting animals and human [6]. They manifest respiratory, gastrointestinal, neurologic, and hepatic diseases [6]. The available statistical data reveal that people >60 years of age and with any morbid conditions have shown higher mortality [5]. The genome of CoVs span between 26 to 32 kb with 6–11 open reading frames (ORFs). The first ORF accommodates 67% of the genome consisting of 16 nonstructural proteins (nsps), while the other ORFs comprise accessory and structural proteins [7]. The three structural proteins that are present on the exterior are the membrane (M), envelope (E), and spike (S), while the nucleocapsid (N) is embedded within the envelop [8]. The main protease (Mpro) performs an essential role in processing the polyproteins of the virus that are instrumental in viral maturation and subsequent infection, thereby making it a promising drug target [9]. Correspondingly, inhibiting Mpro could hinder the viral replication [8], and thus targeting Mpro would be an ideal strategy. Structurally, Mpro consists of three domains, domain I (residues 8–101), domain II (residues 102–184), and domain III (residues 201–303), and a loop (residues 185–200) that connects domain II and domain III [12]. The substrate binding site is present between domain I and domain II, and has a Cys-His catalytic dyad [10]. Mounting evidences report the benefits of targeting Mpro to combat SARS-CoV-2 [10,11,12,13,14].

The DEAD-box (Asp-Glu-Ala-Asp) proteins originate from the RNA helicases and are instrumental in rearranging RNA-RNA and RNA-dependent ATPase. The DEAD-box RNA helicase 3 (DDX3) exists in all eukaryotes [15,16]. Impairment of this DDX3 helicase plays a crucial role in promoting several diseases such as intellectual disability, inflammation, viral infection, and cancer [16]. Specifically, DDX3 has gained interest in the field of cancer biology with its role recorded in around several types of cancers [16]. Accumulating reports evidence the role of DDX3 as a potential target in several viruses [17,18,19]. Finding inhibitors against DDX3 could be a promising therapeutic strategy against different cancers and viral diseases. A recent study has reported that the proteomic analysis has discovered DDX3X as a target for SARS-CoV-2 infection [20].

Structurally, the cocrystallised ligand AMP of DDX3 (PDB code: 2I4I) has shown interactions with the residues arising from the Q motif and the P loop. The adenine group of adenosine monophosphate (AMP) interacts with Arg202 and Gln207, and the phosphate group interacts with the Gly227, Ser228, Gly229, Lys230, and Thr231 residues [21,22].

Computational reports exist on the ability of curcumin to interact with several target proteins of SARS-CoV-2. Jena et al. described the potential of Catechin and curcumin to interact with the S protein of SARS-CoV-2 and ACE2 of human cell membrane [23]. Shanmugarajan et al. have shown that curcumin hinders the binding of spike glycoprotein to ACE2 receptor [24]. Patel et al. described the role of curcumin and its derivatives as SARS-CoV-2 spike protein inhibitors [25]. Additionally, curcumin inhibits SARS-CoV-2 infection in vitro in Vero E6 cells [26]. Kumar et al. targeted curcumin against Nsp9 replicase protein [27]. Liu et al. described that curcumin can be a therapeutic agent against pneumonia and acute lung injury (ALI) or fatal acute respiratory distress syndrome (ARDS) in humans [28]. Thimmulappa et al. reported the role of curcumin role as a potential prophylactic therapeutic against COVID-19 in the clinical and public health settings [29]. Manoharan et al. elucidated curcumin as a wonder drug in COVID-19 management [30]. Likewise, reports exist on the use of curcumin as a potential anticancer treatment [31,32]. Furthermore, reports exist on the ability of curcumin to inhibit the proliferation on several breast cancer cell lines, which includes MCF7, MDA-MB-468, T47D, and MDA-MB-231 [33]. Mohammed et al. determined the anticancer ability of two curcumin complexes, the iron–curcumin (Fe(Cur)_3_) and boron–curcumin (B(Cur)_2_) complexes, in the MDA-MB-231 breast cancer cell line [34]. Our earlier study has revealed that curcumin and exemestane synergistically regulates DDX3 expression in cancer cell lines [35]. 

Motivated by these reports in the current investigation, we have used curcumin analogues against Mpro of SARS-CoV-2 and DDX3 to discover putative candidate inhibitors that have a higher efficacy towards the chosen targets.

## 2. Results

**Scheme 1:**
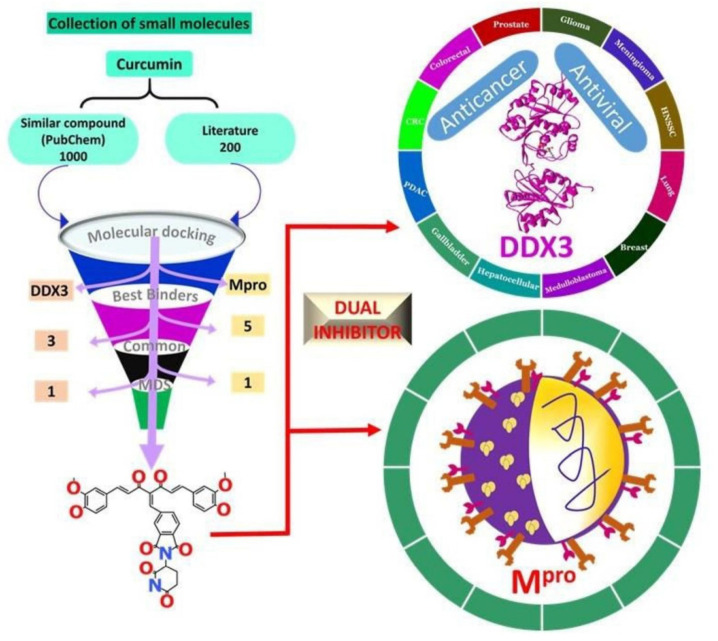
Infographic representation of the work conducted.

A series of computational techniques were used to identify the most potential natural compound analogue, as represented in Scheme 1.

### 2.1. Effect of Natural Compound Analogues on Mpro

#### 2.1.1. Binding Affinity Studies and the Section of the Pose

The molecular docking studies have shown that one compound curA has generated a better dock score than the rest of the compounds complemented by the highest cluster. The cocrystallised structure from the X-ray structure is taken as the reference (ref) compound, (Supplementary Figure S1). The dock score (-CDOCKER interaction energy, kcal/mol) of curA was 58.96 while that of the ref was 56.71. The highest scoring pose from this cluster was chosen as the best pose and was upgraded to MDS to assess the stability during the entire simulation run.

#### 2.1.2. Molecular Dynamics Simulation Studies 

The MDS studies were performed to gain insight on the dynamics of the ligand at the binding pocket of the protein and to examine how the ligand accommodates at the binding pocket.

#### 2.1.3. The Compound Demonstrates a Stable RMSD

**Figure 1:**
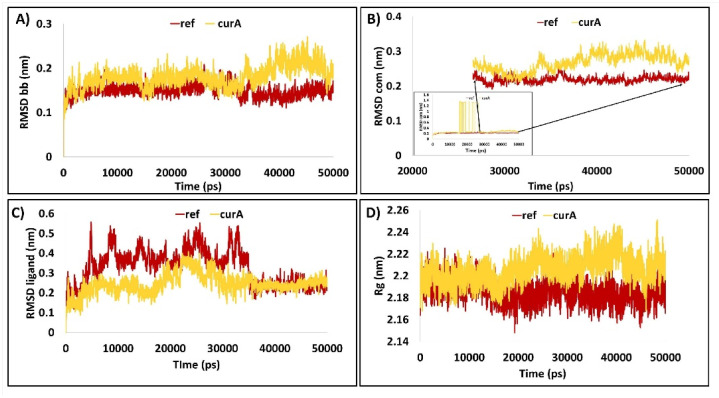
MDS inferred stability results. (**A**) Root mean square deviation of the protein backbone (**B**) RMSD of the protein-ligand complex (com). (**C**) RMSD of the ligand. (**D**) Compactness analysis according to Rg.

During the simulation run, the stability of the protein back bone (bb), protein–ligand complex (com) and the ligand were scrupulously studied. The RMSD of the protein backbone and the protein–ligand complex have rendered an RMSD below 0.3 nm. The reference bb has shown a stable RMSD with an average of 0.14 nm while the average RMSD of curA was about 0.18 nm. Interestingly, the RMSDbb was noted to be deflected at around 34,000 ps for both the systems, which resulted in the marginal rise and decrease in the RMSD of curA and ref, respectively (Figure 1A). The RMSD pattern of the com largely remained the same as that of bb. The ref was well equilibrated and demonstrated a steady profile with an average of 0.21 nm. The results of curA have demonstrated that the bb of the protein has shown a lower RMSD upon comparison with the com. Notably, the RMSD of the complex has shown higher fluctuations between 15,820 ps to 26,590 ps and dropped thereafter, remaining relatively stable (Figure 1B). This deviation is presumed to be due to the ligand that has been adjusting at the binding pocket of the protein. The average RMSD was calculated as 0.21 nm for ref com and 0.38 nm for curA (Figure 1B). After 26,590 ps, the average RMSD of com was recorded as 0.26 nm for curA and 0.21 nm for ref. The RMSD of the ligand has displayed greater fluctuations till 35,000 ps with both the systems and stable thereafter. Higher waves in the profiles were observed with ref upon comparison with curA, which was relatively stable. The overall average of ref was recorded as 0.32 nm, and curA was about 0.24 nm. A high degree of convergence was noticed with ref and curA after 35,000 ps with an average RMSD of 0.23 nm for ref and 0.24 nm for curA (Figure 1C). The RMSD results portray that the profiles were well equilibrated and have demonstrated stable profiles. 

#### 2.1.4. Protein Backbone Is Compact during the Simulation 

The radius of gyration (Rg) talks about the compactness of the protein and was measured for the protein backbone. The Rg of both the systems was found to be ranging between 2.18 nm and 2.24 nm, respectively, determining the compactness of the protein during the simulation (Figure 1D). The average Rg for ref and curA was computed as 2.18 nm and 2.20 nm, respectively. 

#### 2.1.5. The Protein Backbone Has Demonstrated Negligible Fluctuations 

**Figure 2:**
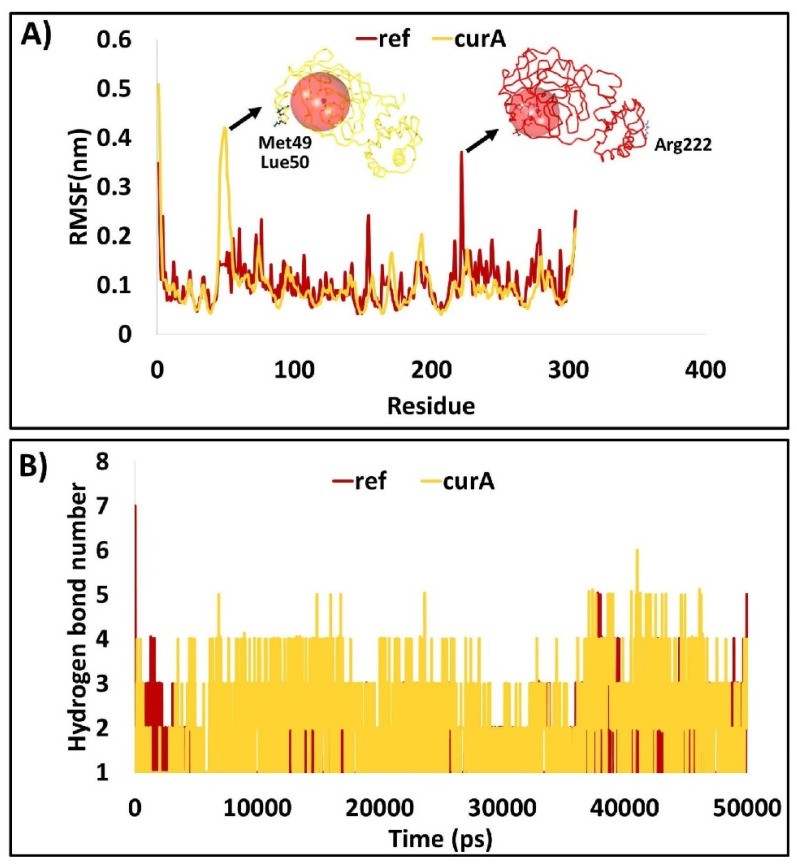
MDS guided results. (**A**) Root mean square fluctuations. The binding pocket is represented in red sphere (**B**) Hydrogen bond number during the simulation run.

The protein backbone residue atoms were measured for any fluctuations by RMSF. The backbone residues have revealed low fluctuations. The RMSF of the residues was projected to be below 0.3 nm, implying that the protein backbone was highly stable, supporting the RMSD and the Rg results. In the bb of ref, a minor peak was noticed with the residue Arg222 when compared with the other residues reaching to about 0.4 nm. However, this residue does not exist in proximity to the binding pocket. In curA, the residues from Met49 and Leu50 have demonstrated a marginal raise that peaked to 0.4 nm. These residues are a part of the loop region that is generally prone to fluctuations (Figure 2A), while the other residues, particularly the active site residues, were found to be stable (Figure 2A).

#### 2.1.6. Ligand Is Accommodated in the Binding Pocket throughout the Simulation Run

**Figure 3:**
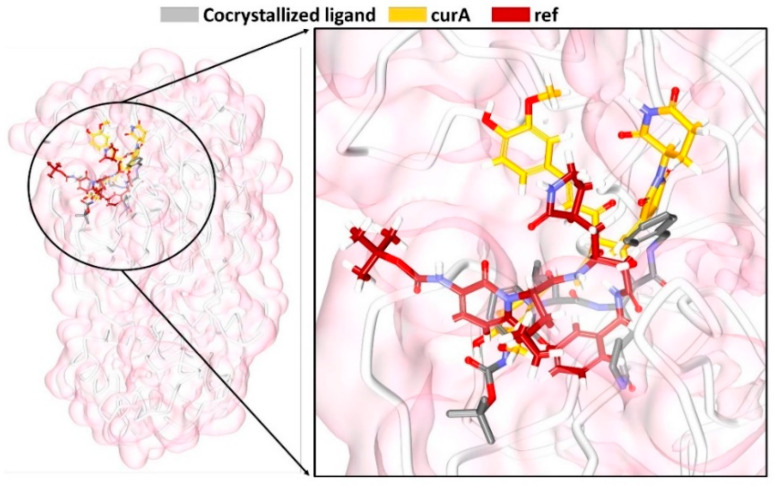
Binding mode analysis. Left panel indicates the accommodation of ligands at the binding pocket of the protein and right panel indicates its zoomed version.

From the last 5 ns of the stable RMSD, the representative structures were extracted and superimposed against the X-ray crystal structure. It was determined that the ref and curA have occupied the same site as that of the cocrystal encircled by various key residues clamping the ligands (Figure 3).

During the simulations, the formation of the hydrogen bonds was observed. Notably, the hydrogen bonds were formed throughout the simulations, suggesting that the ligands remain in the binding pocket during the entire simulation. The average hydrogen bond number for ref was about 0.9, though this figure was potentially greater during the last 10 ns. The curA, on the other hand, has generated more hydrogen bonds throughout the simulation run. The average hydrogen bonds were revealed to be two. It is noteworthy that the hydrogen bonds were recorded to be higher during the last 10 ns with an average of 2.4, implying that the ligand has firmly occupied the active site (Figure 2B). 

**Figure 4:**
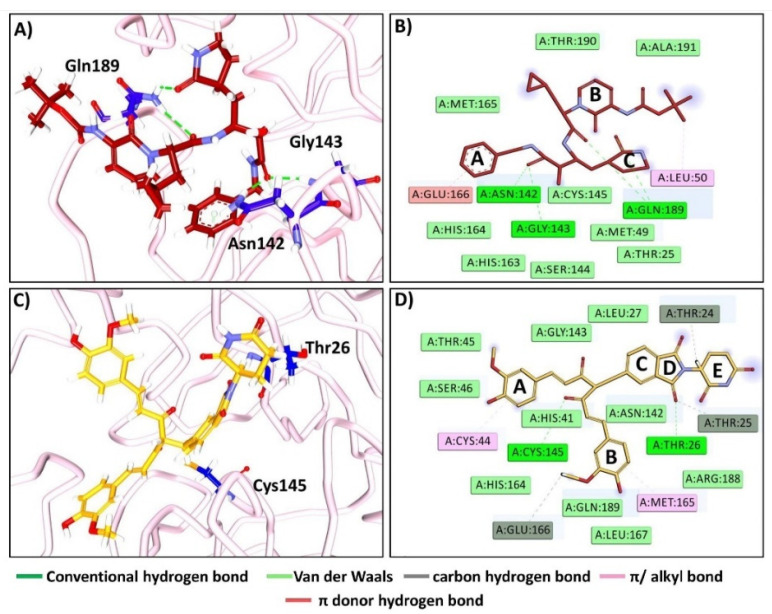
Intermolecular interactions. (**A**) Hydrogen bonds between the protein and ref. (**B**) Comprehensive intermolecular interactions between protein and ref. (**C**) Hydrogen bond interactions between protein and curA. (**D**) Comprehensive intermolecular interactions between protein and curA.

The ref compound has formed hydrogen bonds with Asn142, Gly143, and Gln189, respectively (Figure 4A). Ring A of the ligand has prompted a π donor hydrogen bond interaction with the pivotal residue Glu166 at one end. Leu50 held the other end of the ligand via an alkyl interaction firmly adhering the ligand at the active site of the protein. The other key residues from various subsites have held the ref at the active site of the protein (Figure 4B).

The compound curA has formed two hydrogen bond interactions with the key residues Thr26 and Cys145, measured with an acceptable bond length (Figure 4C). The compound has rendered three carbon hydrogen bonds with the key residues Thr24, Thr25, and Glu166, respectively. Furthermore, ring A is held by Cys44 residue and ring B by Met165 residue, promoting π–alkyl interaction. Additionally, other key residues have accommodated the ligand at the binding pocket, prompted by van der Waals interactions firmly encircling the ligand (Figure 4D).

#### 2.1.7. Distance Measure between the Interacting Residues and the Ligand Atoms 

**Figure 5:**
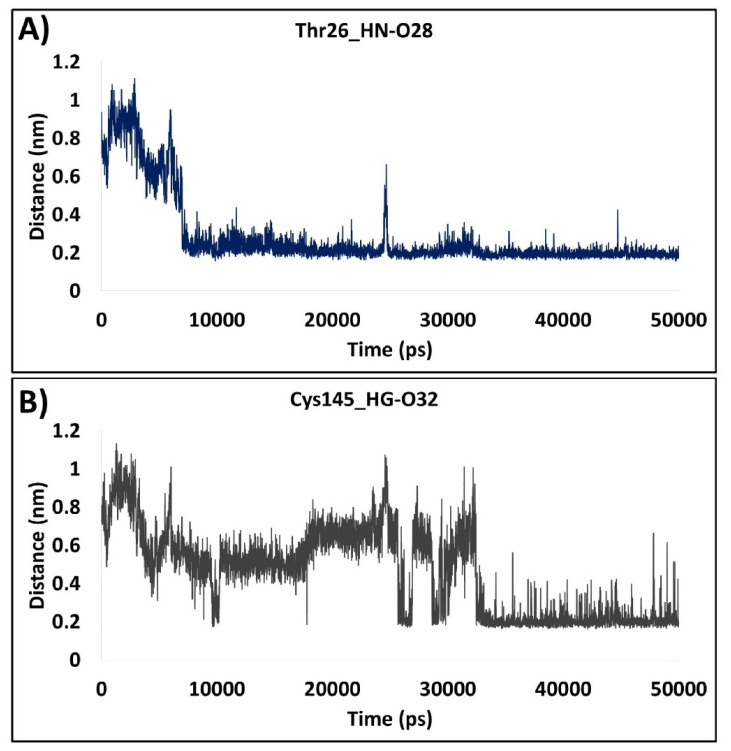
Hydrogen bond distance measure. (**A**) Distance between Thr26 and its corresponding ligand atom. (**B**) Distance between Cys145 and its corresponding ligand atom.

To understand the hydrogen bond interactions during the evolution of the simulation, we have calculated the distance measure of the two hydrogen bonds formed between the target and curA. The distance between Thr26_HN-O28 was a seemingly a rigid interaction with an overall average of 0.28 nm. Although it showed a higher distance during the initial simulation runs until 8000 ps, it significantly dropped after 8000 ps and remained relatively stable thereafter (Figure 5A). The interaction between Cys145_HG-O32 showed a high degree of variation ranging between 0.2 nm and 1.0 nm. Interestingly, the fluctuations were observed until 32,000 ps with an overall distance of 0.4 nm. After 32 ns, the distance plot remains largely stable with an average of 0.22 nm (Figure 5B). This finding deduces that the Thr26_HN-O28 interaction was highly stable and firmer than Cys145_HG-O32, which gained stability after 32 ns. The variations can be inferred because of the adjustment of the ligand at the active site of the protein.

#### 2.1.8. The Protein–Ligand Complex Projects the Better Interaction Energy

**Figure 6:**
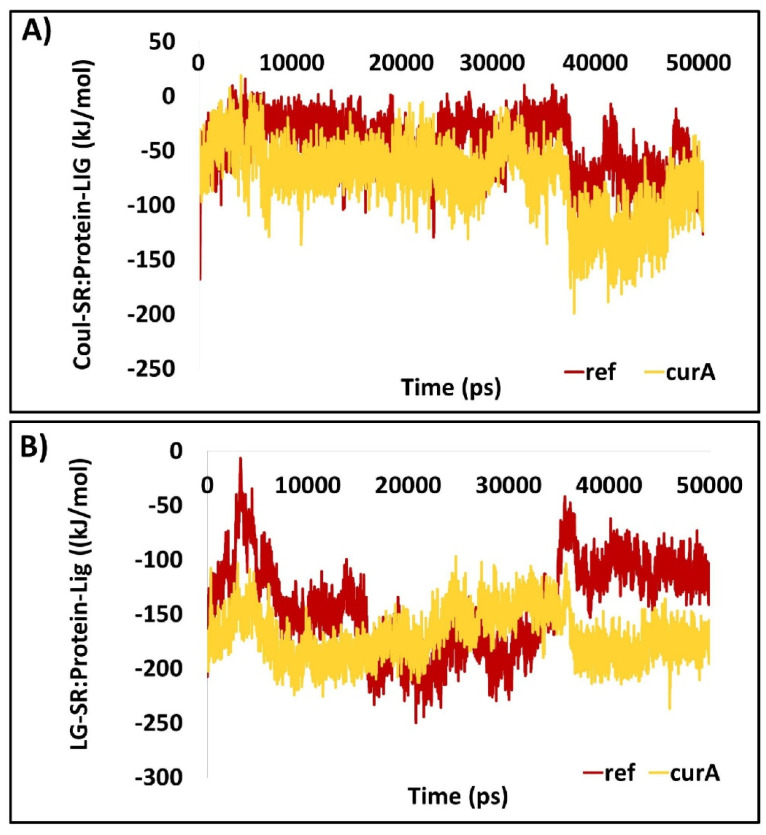
Interaction energy (IE) between protein and ligands. (**A**) Coulombic IE (**B**) Lennard-Jones IE.

The interaction energy (IE) infers to the binding potential of the ligand at the active site protein and was calculated according to Coulombic interaction energy (CIE) and Lennard-Jones IE (LJIE), respectively. The CIE of ref and curA has ranged between −50 kJ/mol and −175 kJ/mol with an average of −44.05 kJ/mol for ref and −75.47 kJ/mol for curA, respectively. The pattern of CIE plots was similar for both ref and curA with a drop observed at 36,720 ps (Figure 6A). The LJIE was computed to be between −50 kJ/mol and −250 kJ/mol for ref and curA with an average of −141.96 kJ/mol for ref and −170 kJ/mol for curA (Figure 6B). Both the interaction energies make it reasonable to presume that the ligand interacts with the protein (Figure 6).

#### 2.1.9. Essential Dynamics and Free Energy Landscape

**Figure 7:**
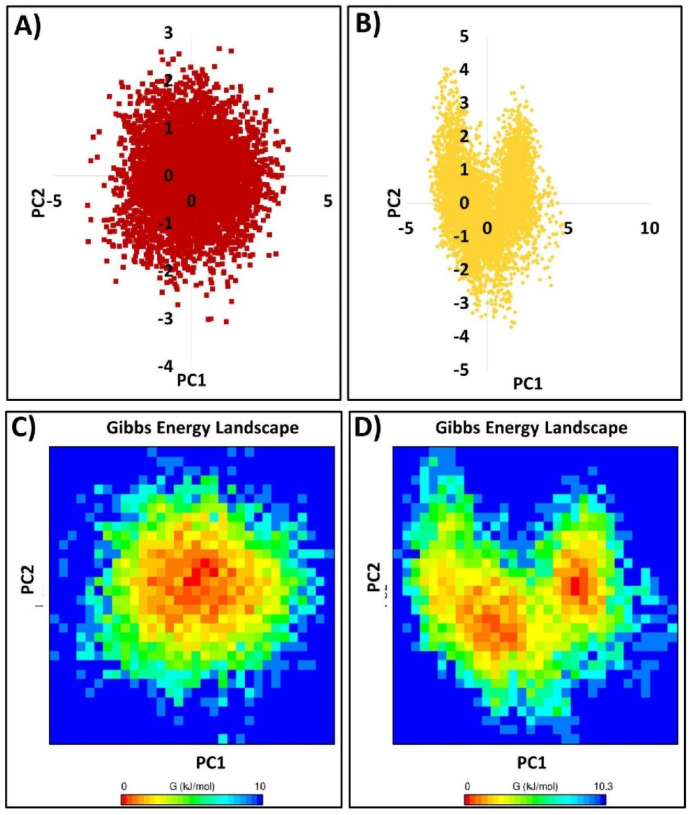
Insights from essential dynamics (ED). (**A**) PC analysis of ref. (**B**) PC analysis of curA. (**C**) Free energy landscape of ref. (**D**) Free energy landscape of curA. The colour code designates the confirmations from favourable to unfavourable conformations.

The function of the proteins is governed by different conformational changes and plays a pivotal role in the transmission of biological signals [36]. Additionally, the flexibility and the rigidity of a protein, particularly to that of the residues in the binding pocket, attribute a protein to be functional. To gauge the overall movement of the protein, the essential dynamics (ED) were applied for the first two PCs, PC1 and PC2, and were enumerated by diagonalizing the covariance matrix of eigenvector to mark the important subspaces where the major protein dynamics would occur. From the PC analysis, it was evident that the overall motion was minimum for ref (Figure 7A), as was noticed with the other structural analysis results such as RMSD and Rg. Interestingly, the curA protein was noticed to navigate through a marginal range of conformational spaces in PC1 prior to reaching an equilibrated state (Figure 7B). The ED directs to the fact that the protein has undergone an appropriate flexibility to attain an equilibrated state. Overall, it can be postulated that the protein has undergone minimal conformational changes before reaching the equilibrated state. The binding of the curA to the target was attained by two minimum energy basins, as represented by red to green colours (Figure 7D), dissimilar to the ref (Figure 7C). Furthermore, the protein has shown larger favourable conformations, as represented by the colour code from red to light green and further to blue (Figure 7C,D).

### 2.2. Effect of Natural Compound Analogues on DDX3

#### 2.2.1. Binding Affinity Studies

The binding affinity studies between DDX3 and curcumin analogues have yielded one compound as the best compound rendered from the largest cluster complemented by the key residue interactions. The RK-33 has been taken as the reference (ref) compound for superlative analysis, (Supplementary Figure S1). The compound, curA, has demonstrated a dock score of 52.36 (-CDOCKER interaction energy, kcal/mol) encircled by the vital residues holding the ligand firmly at the binding pocket. The reference compound has projected a score of 50.75 kcal/mol. To delineate on the stability of the compound at the binding pocket, the complex was upgraded to MDS studies.

#### 2.2.2. Molecular Dynamics Simulation Studies

To understand in detail the stability of the complex, the MSD was initiated. This technique is paramount to gaining knowledge about the macromolecular structure–function relationship [37]. 

#### 2.2.3. Stability Analysis by Root Mean Square Deviation

**Figure 8:**
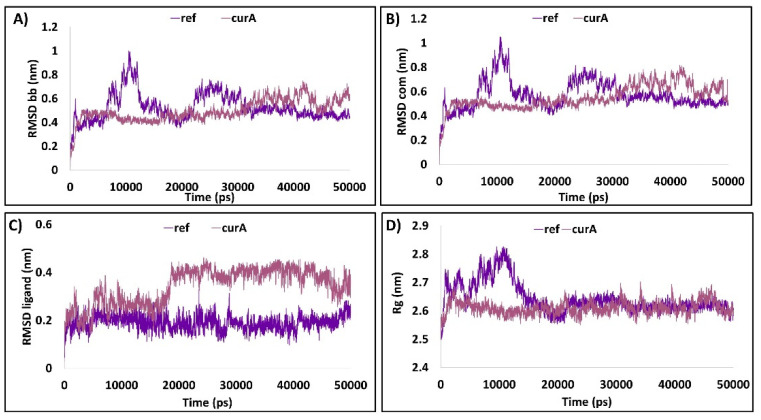
MDS inferred stability results. (**A**) Root mean square deviation of the protein backbone (**B**) Root mean square deviation of protein–ligand complex. (**C**) RMSD of the ligand. (**D**) Compactness of the protein backbone according to Rg.

RMSD refers to the occurrence of deviations that were noticed during the evolution of the simulation [38,39,40]. Furthermore, the RMSD defines the stability of the protein as the smaller the RMSD, the greater the stability [39]. In the current investigation, the RMSD was examined for the protein backbone (bb), ligand, and protein–ligand complex (com). The ref bb demonstrated an average RMSD of 0.52 nm with an initial raise in the RMSD during 6000 ps to 13,210 ps, which remained stable thereafter. The curA bb was well equilibrated from the origin of the simulation and remained stable during the progression of the simulation with an average of 0.49 nm (Figure 8A). Interestingly, the protein–ligand complex also generated a similar profile, suggesting that the ligand must have resided in the binding pocket of the protein during the advancement of the simulation (Figure 8B). Likewise, the ligand also demonstrated a stable RMSD during the entire simulation. Notably, the ref was stable with an average of 0.19 nm, while the curA showed a marginal raise at 17,500 ps and then remained stable (Figure 8C). 

#### 2.2.4. Radius of Gyration

The radius of gyration (Rg) governs the protein compactness. The average Rg for ref was recorded as 2.64 nm. The Rg profile for the ref was observed to be raised from 6000 ps to 14,360 ps, as was observed with the RMSD, after which the system was finely equilibrated during the process of simulation (Figure 8D). On the other hand, the protein backbone of curA was remarkably compact during the course of the simulation with an average of 2.60 nm (Figure 8D). The RMSD and the Rg findings highlight that the protein structure was steady during the simulation run (Figure 8).

#### 2.2.5. Root Mean Square Fluctuations

**Figure 9:**
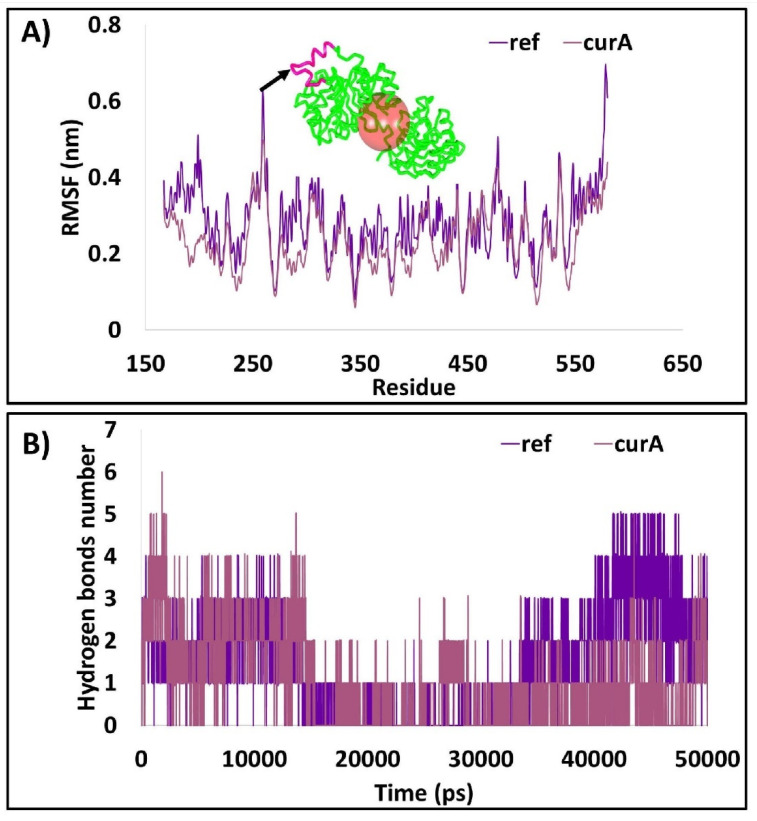
Molecular dynamics simulation inferred findings. (**A**) Fluctuations in the residues during the simulation run. The residues with high fluctuations are shown in pink, which are away from the binding site indicated in red sphere. (**B**) Number of hydrogen bond interactions during the evolution of the simulation.

RMSF plots impart knowledge on the residue specific fluctuation rendered during the simulations. The results of ref and curA have shown that, largely, the protein was stable with no major fluctuations. However, the residues from 250–266 have demonstrated fluctuations of about 0.4 nm. Interestingly, these residues are not part of the binding pocket and therefore, it is speculated that they have no major impact on the binding of the ligand (Figure 9A). 

#### 2.2.6. The Compound Accommodates at the Same Binding Pocket as the Cocrystallised Ligand

**Figure 10:**
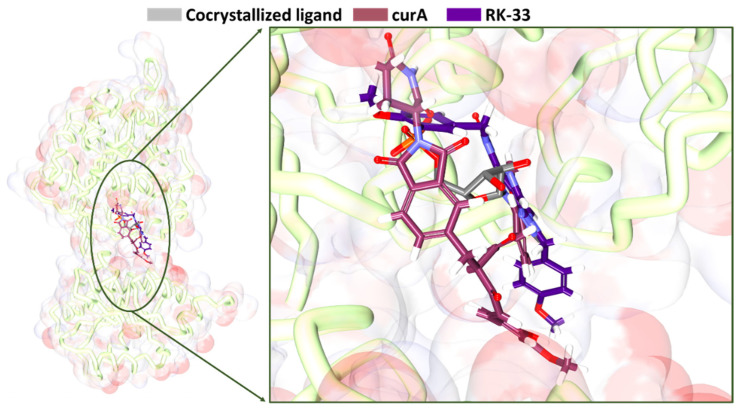
Binding mode analysis. Left panel infers ligand is seated at the same binding pocket as that of the cocrystallised ligand. Right panel is the zoomed version.

**Figure 11:**
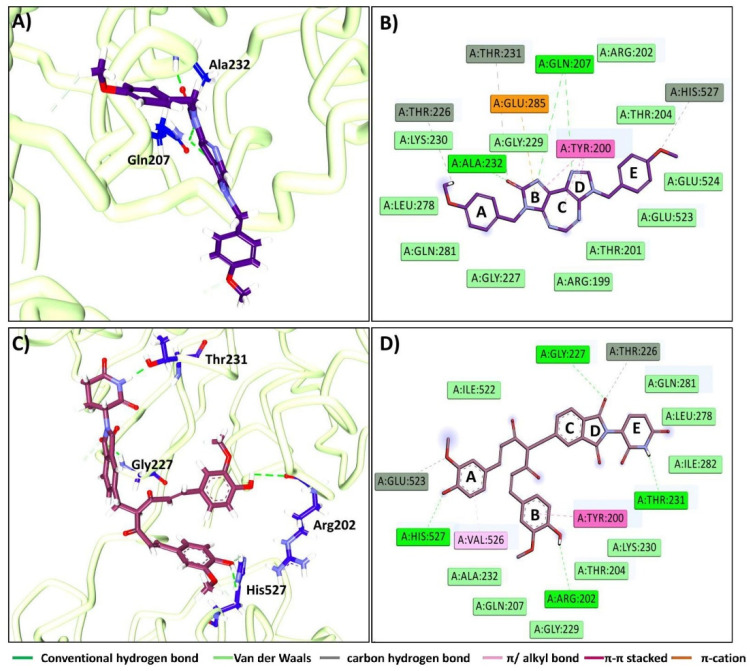
Interaction between the protein residues and the ligand. (**A**) Hydrogen bond interactions between protein and ref. (**B**) Comprehensive intermolecular interactions between protein and ref. (**C**) Hydrogen bond interactions between protein and curA. (**D**) Comprehensive intermolecular interactions between protein and curA.

From the stable RMSD, the last 5 ns structures were extracted and aligned on to the X-ray structure. It was revealed that the compound was positioned at the same binding pocket as that of the cocrystallised ligand clamped by various residues (Figure 10). The ref has generated two hydrogen bonds with the key residues Gln207 and Ala232, respectively (Figure 11A). Interestingly, the key residue Tyr200 has prompted a π–π stacked interaction with the ring B, C, and D of the ligand. Ring B has additionally formed a π–interaction with the residue Glu285. The peripheral region of the ref was held by the carbon hydrogen bonds with the residues Thr226 and His 527 accommodating the ligand at the binding pocket of the protein. The other key residues have generated van der Waals interactions, adhering the compound at the binding pocket (Figure 11B).

The compound curA has formed four hydrogen bond interactions with the residues Arg202, Gly227, Thr231, and His527, respectively (Figure 11C). The key residue Tyr200 has interacted with ring B of the ligand via π–π stacked interaction. The residue Val526 has generated a π–alkyl interaction with ring A of the ligand. Other key residues have clamped the ligand with van der Waals interaction firmly holding the ligand at the active site of the protein (Figure 11D). 

#### 2.2.7. Distance Measure between the Interacting Residues and the Ligand 

**Figure 12:**
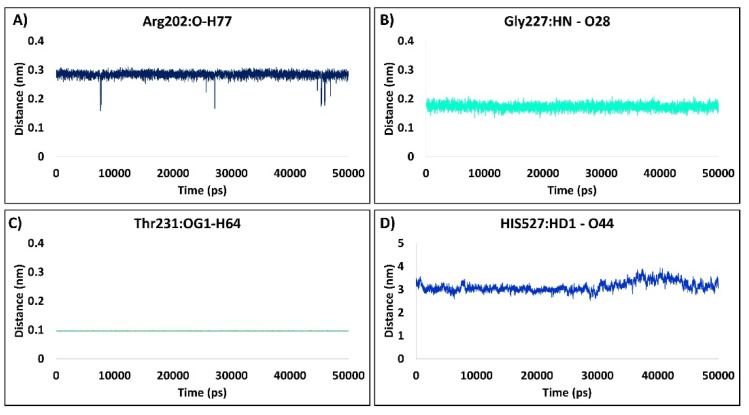
Distance measure between the hydrogen bond forming residues and the ligand atoms. (**A**) Arg202 (**B**) Gly227 (**C**) Thr231 (**D**) His527.

During the evolution of the simulation run, the distance between the residues forming the hydrogen bonds and their ligand counterpart atoms was determined for curA. A detailed analysis has revealed that the three hydrogen bonds, Arg202, Gly227, and Thr231, were strong with an average of 0.3 nm, while the His527 was weak as inferred from the distance analysis (Figure 12). The residue Arg202 has formed a stable interaction throughout the simulation, illuminating its strong binding with an average of 0.28 nm (Figure 12A). The key residue Gly227 has formed a persistent interaction with the ligand atom with an average of 0.17 nm with no deviations noticed during the length of the simulation (Figure 12B). Of all the interactions, the residue Thr231 has promoted a stronger interaction with an average of 0.09 nm observed consistently during the progression of the simulation (Figure 12C). The interaction of the ligand with His527 was stable between 3 and 3.5 nm with an average of 3.12 nm (Figure 12D). It is noteworthy that this residue is not a reported key residue. From this analysis, it can be deduced that the ligand was held within the binding pocket, forming the stable interactions with the key residues and further complemented by other residues forming van der Waals interactions.

#### 2.2.8. The Protein–Ligand Complex Projects the Better Interaction Energy

**Figure 13:**
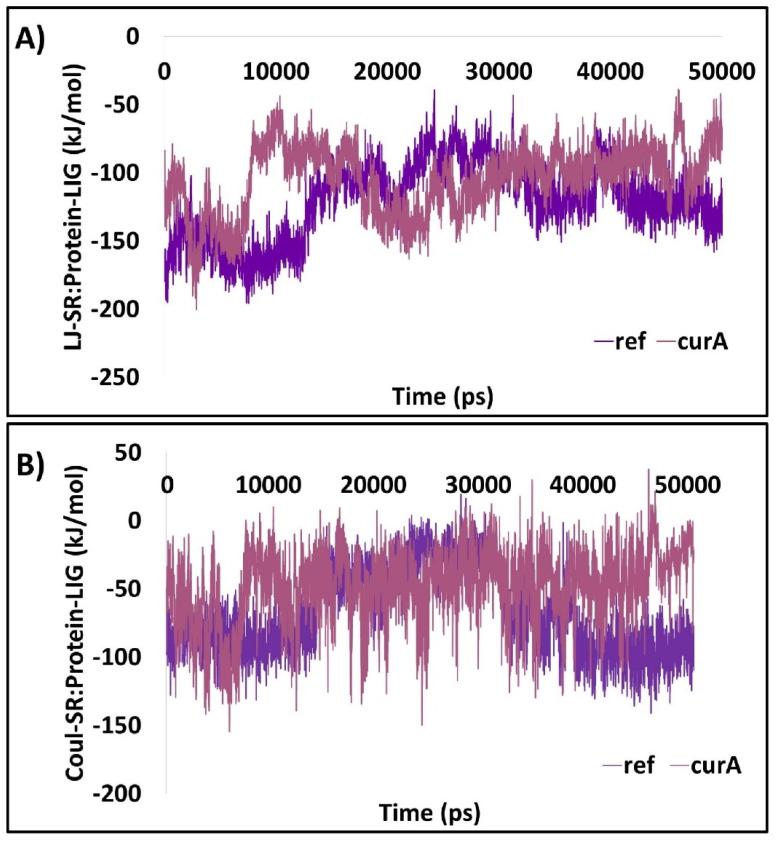
Interaction energy (IE) between protein and ligands. (**A**) Lennard-Jones IE. (**B**) Coulombic IE.

The interaction energy (IE) of the ligand at the proteins active site were computed according to CIE and LJIE, respectively. The LJIE was computed to be between −50 kJ/mol and −200 kJ/mol, with an average of −120.86 kJ/mol for ref and −104.80 kJ/mol for curA (Figure 13A). The CIE of ref and curA has ranged between −10 kJ/mol and −160 kJ/mol with an average of −66.51 kJ/mol for ref and −48.41 kJ/mol for curA, respectively (Figure 13B). Both the interaction energies make it reasonable to assess that the ligand interacts with the protein (Figure 13).

#### 2.2.9. Essential Dynamics

**Figure 14:**
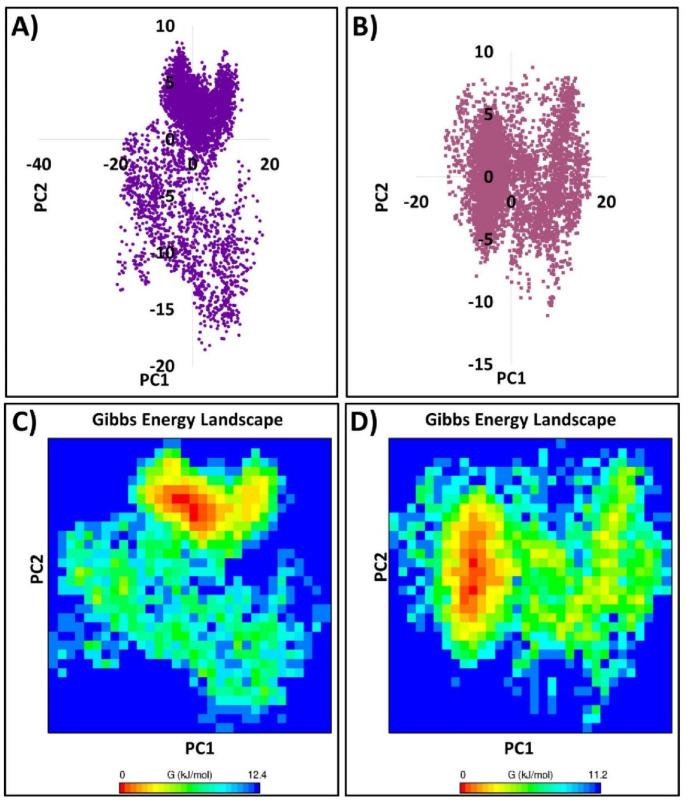
Essential dynamics studies. (**A**,**B**) represent the PC analysis of ref and curA (**C**,**D**) denotes the free energy landscape of ref and curA. The colour code indicates the favourable conformation to unfavourable conformations.

To gain deeper insight into the dynamic motion of the protein, the essential dynamics evaluation is an important parameter. Generally, proteins exhibit some amount of flexibility and rigidity, in particular to the residues located at the binding pocket. Correspondingly, to comprehend the overall motion of the protein demonstrated during the simulation run, the dimension reduction method, essential dynamics (ED), was conducted for the first two principal components (PCs). The diagonalization of the covariance matrix of eigenvectors was performed to effectively expound the subspaces where the protein dynamics occurs. The results have shown that the protein has navigated widely through PC1 prior to reaching the equilibrated state in the case of ref, and PC2 in the case of curA (Figure 14A,B). This is also reflected from the free energy landscape. This analysis shows that the protein has travelled though different energy basins displayed by favourable conformations (red to yellow colour) (Figure 14C,D)

## 3. Discussion

Natural compound derivatives have demonstrated an imperative role in the drug discovery process, especially for cancer and infectious diseases [41]. Typically, natural products have diverse scaffolds, possess higher molecular mass, have a greater number sp^3^ carbon atoms and oxygen atoms, and less halogen and nitrogen atoms [41]. The number of H-bond acceptors and donors are high with low computed octanol–water partition coefficients accompanied by favourable molecular rigidity, suggesting that the natural products are enriched with several bioactive compounds [41]. 

With an aim to discover new natural compound derivatives as plausible inhibitors for SARS-CoV-2, we have considered the curcumin analogues. Earlier reports have suggested that curcumin has a potential to treat COVID-19 and cancer [28,29,32,42,43]. Encouraged by these reports, we have used curcumin analogues for unravelling the prospective candidate compound.

The chosen compound curA is a thalidomide–curcumin hybrid essentially designed to elevate the biological properties of thalidomide [2]. Their antiproliferative activities were evaluated against human multiple myeloma MM1S, RPMI8226, U266 cells, and human lung cancer A549 cells. Notably, the curA has remarkably amplified their effect in all the cell lines upon comparison with the curcumin or thalidomide alone and their combination [2].

The molecular dock scores have indicated one compound as the best compound and its stability at the protein’s binding pocket was explored in detail. The MDS has revealed that curA has accommodated at the binding pocket of Mpro, aided by several vital residues. Particularly, the compound has formed a stable interaction with Thr26 and catalytic dyad Cys145 residue. Interactions with these residues were reported in previous studies [12,44,45]. The catalytic dyad His41 has prompted a van der Waals interaction, illuminating the ability of curA to be effective against SARS-CoV-2. In addition, CurA has formed several key interactions with crucial residues from various subsites, fully occupying the active site (Figure 3). The MDS have affirmed the usability of curA against COVID-19. These results have demonstrated that curA has firmly occupied the binding site during the course of the simulation. The hydrogen bond distance plots denote that the residue Thr26 has firmly adhered with the curA during the evolution of the simulation, more so than the residue Cys145. The key residue Cys145 produced a stronger interaction from 35,000 ps. The interactions with these residues were previously reported with varied compounds [45,46,47,48]. These findings provide substantial evidence to endorse curA as a plausible inhibitor for COVID-19. Additionally, the stability analysis examined according to RMSD and Rg have specifically conferred that the protein–ligand complex was relatively stable and compact (Figure 1). This finding was in agreement with the PCA result. Interestingly, the protein has generated fewer unfavourable conformations, with a greater number of favourable conformations through which the interaction with the ligand must have taken place. The sum of all these findings are suggestive that curA can be used for SARS-CoV-2 treatment regime after sufficient wet lab and clinical trials. 

The binding affinity studies have shown that curA has a better affinity towards the target DDX3 than the ref. Furthermore, the compound has stationed at the binding pocket by interacting with the key residues Arg202, Gly227, and Thr231, respectively, during the simulation run. Interactions with these residues were noticed with the X-ray crystal structure and the previously reported studies [49,50]. Interestingly, the His527 residue was also observed in a previous study [51]. Furthermore, the MDS results have confirmed that the RMSD and Rg results were relatively stable during the simulation run, elucidating the accommodation of ligand within the binding pocket. The same was reflected by the PC analysis. These results support the use of curcumin analogue for DDX3 triggered cancers and also as an antiviral agent. 

The detailed computational analysis conducted in the current study to retrieve a putative natural compound analogue has discovered curA as a possible dual inhibitor as an antiviral and anticancer inhibitor. Furthermore, this compound may be used as an initial structure to design and develop new inhibitors. 

## 4. Materials and Methods

### 4.1. Target Structure

The viral protein, the main protease (Mpro, PDB code: 6Y2F), and human DDX3 (PDB code: 2I4I) were chosen for the current investigation. The proteins were prepared by enabling the “*clean protein*” tool accessible with the Discovery Studio v18 (hereinafter referred to as DS). This process was undertaken to evaluate for the presence of any gaps or missing residues. The water molecules and the hetero atoms were removed and the protein was subsequently minimized. Correspondingly, the active site was secured for all the atoms and residues that fall within the radius of 10 Ǻ.

### 4.2. Selection of the Small Molecules

The curcumin and its analogues were chosen for the present investigation as reported earlier in the literature [2,52,53]. The compounds were sketched on *Biovia Draw* in the 2D, *.mol format and were escalated to DS to generate the 3D structure. All the compounds were minimized enabling the “*Full Minimization*” protocol available with the DS.

### 4.3. Molecular Docking to Predict the Binding Mode of the Ligands

The CDOCKER programme [54] accessible with the DS was used to effectively predict the binding modes of the compounds complemented by the favourable -CDOCKER interaction energy. For every ligand, 50 poses were generated and subsequently clustered. From the largest cluster, the best binding pose was selected to delineate the atomic interactions. The simulated heating step was selected as 2000, heating target temperature was 700, cooling step was 5000, and cooling target temperature was 300.

### 4.4. Molecular Dynamics Simulation (MDS) Studies

The MDS studies were undertaken to understand the stability of the protein–ligand complex. For its accomplishment, the GROMACS v2016.16 [55], using CHARMM 27 all atom force field, was used. The ligand topologies were obtained from SwissParam [56]. The simulation run was conducted as explained earlier [57,58]. A dodecahedron water box was generated and solvated with TIP3P water model. This was followed by the addition of counter ions and the system was subsequently minimized with steepest descent minimization algorithm. The protein and the ligand were then coupled followed by the double step equilibration method. The first step was with NVT (fixed number of particles (N), system volume (V), and temperature (T)) and the second step was NPT (fixed number of particles (N), system pressure (P), and temperature (T)). The V-rescale modified Berendsen thermostat was used. The NVT and the NPT equilibration were executed for 1 ns each and the MD run was conducted for 50 ns under periodic boundary conditions. The remaining parameters were set at default. The results were analysed on DS, visual molecular dynamics (VMD) [59]. The stability analysis was studies according to the root mean square deviation (RMSD), root mean square fluctuation (RMSF), radius of gyration (Rg), interaction energy, number of hydrogen bonds, and their distance.

## 5. Conclusions

Natural compounds have been at forefront in providing medical formulations since the ancient times. Curcumin is widely known for its vast therapeutic potential, in addition to being used as spice. In the current investigation, a wide number of curcumin analogues have been utilized against SARS-CoV-2 target, Mpro, and human DDX3, using diverse computational approaches. The discovered curA has shown promising MDS results, complemented by the stable hydrogen bond interactions during the progression of the simulations. Together, these in silico findings support the usability of curA as a plausible dual inhibitor for SARS-CoV-2 and DDX3.

## Data Availability

Not applicable.

## Supplementary Materials

The following supporting information can be downloaded at: https://www.mdpi.com/article/10.3390/ijms23031771/s1.
