# Supplementary material for: Molecular Docking and Molecular Dynamics Simulations Discover Curcumin Analogue as a Plausible Dual Inhibitor for SARS-CoV-2
Source: Int J Mol Sci. 2022 Feb 4;23(3):1771. doi: 10.3390/ijms23031771 (PMC8836015; doi:10.3390/ijms23031771)
Supplement: Supplementary file 1 [file ijms-23-01771-s001.zip › ijms-1561903-supplementary.pdf]

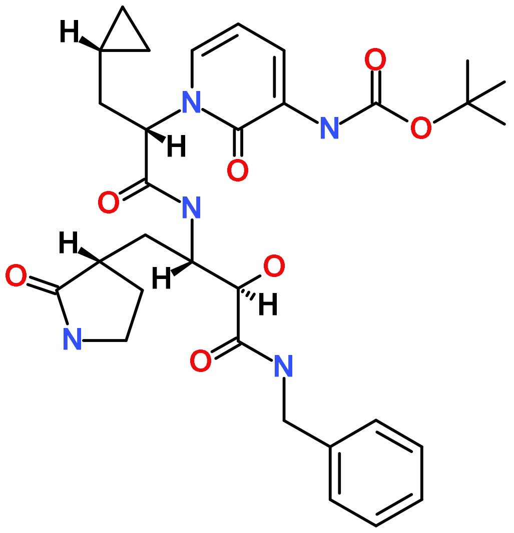

O=C(OC(C)(C)C)N/C1=C/C=CN(C1=O)[C@@]([H])(C[C@@]2([H])CC2)C(=O)N[C@@]([H])(C[C@]3([H])CCNC3=O)[C@](O)([H])C(=O)NCc4ccccc4 6Y2F

IUPAC NAME: tert-butyl N-[1-[(1S)-2-[[(1S,2R)-3-(benzylamino)-2-hydroxy-3-oxo-1-[[(3S)-2-oxopyrrolidin-3-yl]methyl]propyl]amino]-1-(cyclopropylmethyl)-2-oxo-ethyl]-2-oxo-3-pyridyl]carbamate

Reference (ref) compound for Mpro

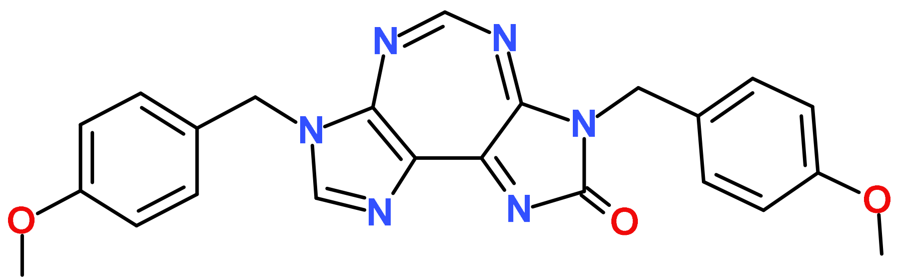

COc1ccc(CN2C(=O)N=C3C2=NC=Nc4c3ncn4Cc5ccc(OC)cc5)cc1 RK-33

IUPAC NAME: 5,11-bis[(4-methoxyphenyl)methyl]-3,5,7,9,11,13-hexazatricyclo[8.3.0.0^{2,6}]trideca-1(10),2,6,8,12-pentaen-4-one

Reference (ref) compound for DDX3

Supplementary Figure S1. 2D structures for ref compounds.
